# Depression symptom and professional mental health service use

**DOI:** 10.1186/s12888-015-0646-z

**Published:** 2015-10-24

**Authors:** Jeong Lim Kim, Jaelim Cho, Sohee Park, Eun-Cheol Park

**Affiliations:** 1 Department of Public Health, Yonsei University College of Medicine, Seoul, Republic of Korea; 2 Institute of Health Services Research, Yonsei University, Seoul, Republic of Korea; 3 Department of Occupational and Environmental Medicine, Gachon University Gil Medical Center, Incheon, Republic of Korea; 4 Department of Biostatistics, Graduate School of Public Health, Yonsei University, Seoul, Republic of Korea; 5 Department of Preventive Medicine & Institute of Health Services Research, Yonsei University College of Medicine, 50 Yonsei-ro, Seodaemun-gu, Seoul, 03722 Republic of Korea

**Keywords:** Mental health, Depression symptoms, Professional mental health consultation, Psychiatric services, Underutilization

## Abstract

**Background:**

Despite the sharp rise in antidepressant use, the underutilization of mental healthcare services for depression remains a concern. We investigated factors associated with the underutilization of mental health services for potential depression symptoms in the Republic of Korea, using a nationally representative sample.

**Methods:**

Data were obtained from the Community Health Survey (2011–2012) conducted in the Republic of Korea. Participants comprised adults who reported potential depression symptoms during the year prior to the study (*n* = 21,644); information on professional mental healthcare use for their symptoms was obtained. The association of demographic, socioeconomic, and health-related factors with consultation use was analysed via multiple logistic regression. Adjusted odds ratio and 95 % confidence intervals were estimated.

**Results:**

Among those reporting potential depression symptoms, only 17.4 % had consulted a medical/mental health professional. Elderly individuals of both genders had significantly lower consultation rates compared to middle-aged individuals. Unmet healthcare needs and a history of diabetes mellitus were associated with lower consultation rates. After stratification by age, elderly individuals with the lowest education and income level were significantly less likely to seek professional mental health services. Married, separated, or divorced men had lower consultation rates compared to unmarried individuals, whereas married, separated, or divorced women had higher rates.

**Conclusions:**

The results suggest that target strategies for vulnerable groups identified in this study—including elderly individuals—need to be established at the community level, including strengthening social networks and spreading awareness to reduce the social stigma of depression.

**Electronic supplementary material:**

The online version of this article (doi:10.1186/s12888-015-0646-z) contains supplementary material, which is available to authorized users.

## Background

It is estimated that almost 350 million people suffer from depression globally [1]. Unipolar depressive disorder—the third leading cause of disease burden in 2004— is expected to become the leading cause of disease burden worldwide by 2030 [2]. Depression may result in poorer well-being [3], and is a predictor of suicidal behaviours, along with affective temperament and hopelessness [4]. Despite the drastic increase in antidepressant use since the 1990s in Western countries [5, 6], elderly individuals’ underutilization of mental health services for depression remains a concern [7, 8]. In Asian countries, people with depressive symptoms are less likely to visit mental health professionals due to the stigma of mental illness [9–11]. However, few studies have examined mental health service use among Asians. In order to develop prevention strategies for depression and suicide, factors associated with the underutilization of mental health services need to be identified.

Among determinants related to mental health service use, previous studies on determinants of mental health service use have focused on help-seeking attitudes, finding that elderly individuals showed more positive attitudes than younger individuals did [12–14]. A population-based study showed that those with lower education and income had negative help-seeking attitudes for mental illness [13, 15]. However, the age difference in the association between socioeconomic status and help-seeking attitudes or behaviours for mental illness has rarely been evaluated. Older adults—despite their relatively positive help-seeking attitudes—are less likely to have access to resources including mental health professionals [16, 17] and rarely consult mental health professionals, compared to middle-aged individuals [7]. Particularly in the Republic of Korea, there has been growing concern about elderly individuals’ mental health problems because of the steep rise in suicide rates, from 42.0 per 100,000 habitants in 2001 to 81.9 in 2010 [18]. Thus, socioeconomic and health-related factors associated with the underutilization of mental health services need to be explored with a focus on age differences.

In the present study, we focused on depression symptoms, investigating demographic, socioeconomic, and health-related factors associated with the underutilization of professional mental health services based on a representative community sample. Additionally, we conducted identical analyses after stratification by age and gender to assess effect modification of the association between socioeconomic and health-related factors and professional mental health service use.

## Methods

### Participants

The Community Health Survey (CHS), a nationwide cross-sectional survey, was conducted by the Korea Centers for Disease Control and Prevention. A systematic, stratified, and multi-stage cluster sampling was performed using the National Census Registry data. The weight assigned to each participant was calculated based on geographic and demographic distribution to allow the extrapolation of findings for the entire population of the Republic of Korea. From the total of 458,147 survey participants (see Additional file 1: Table S1), the total number of participants was 423,106 (209,660 in 2011 and 213,446 in 2012) after excluding missing values.

All participants in the CHS were asked, ‘Were you feeling sad or hopeless for two consecutive weeks during the past year, such that you had difficulty performing your usual activities?’ Only those who responded ‘Yes’ were considered to show ‘potential depression symptoms’ (*n* = 23,625) and were included in the present study. We obtained the permission to use the CHS data from Korea Centers for Disease Control and Prevention through the CHS website (https://chs.cdc.go.kr/chs/index.do) [19].

### Measurement

For the CHS, participants reporting potential depression symptoms were asked, ‘Have you ever consulted professionals (e.g. medical doctors, professional counsellors) for the depressive symptoms?’ In the present study, professional mental health service use or consultation was determined by their ‘yes’ or ‘no’ response.

Participants were grouped according to age: young adults (19–44 years), middle-aged individuals (45–64 years), and elderly individuals (65 years and older) [20]. Socioeconomic factors included education level (primary or less, middle school, high school, and college or more), marital status (married, separated/divorced, and unmarried), income (quartiles 1–4, determined by household income: monthly individual income divided by the square root of household size) [21], residence (metropolitan, urban, and rural), and employment status (unemployed and employed). Health-behaviour factors included smoking status (current, former, and non-smoker), drinking status (drinker and non-drinker), physical activity (‘yes’ or ‘no’), and sleep (<6, 6–9, and ≥9 h per day) [22]. Health-condition factors included subjective health status (good, moderate, and poor), perceived stress susceptibility (very high, high, low, and very low), unmet healthcare needs (‘yes’ or ‘no’), and a medical history (any previous diagnosis of hypertension, diabetes mellitus, hyperlipidaemia, cardiovascular disease [CVD; including angina, myocardial infarction, and stroke], arthritis, and asthma).

### Statistical analysis

Data were analysed using SAS version 9.3 (SAS institute, Cary, NC). Data with missing values in any question were excluded (*n* = 1,981). Thus, 21,644 participants were included in the statistical analysis. Weighted frequency was estimated using ‘PROC SURVEYFREQ’ for categorical variables. Chi-squared tests were used to determine the significance of differences between participants who did and did not receive professional mental health consultation for potential depression symptoms. Multiple logistic regression was used to obtain adjusted odds ratios (ORs) and 95 % confidence intervals (CIs) using ‘PROC SURVEYLOGISTIC’. In addition, the identical analysis was conducted after stratification by gender and age. All statistical tests were two-tailed, and statistical significance was set at *p* < 0.05.

## Results

The study population’s general characteristics and utilization of professional mental health services for potential depression symptoms are summarized in Table 1. Among the 21,644 participants who reported potential depression symptoms, 3,762 (17.4 %) had consulted a mental health professional for their symptoms. The proportions of those seeking professional mental healthcare differed significantly for all variables except marital status (*p* = 0.393) and physical activity (*p* = 0.348).

**Table 1:** Proportion of participants reporting potential depression symptoms who consulted a mental health professional

Characteristics		Total				Men				Women			
		*N* = 3762/21,644 (17.4 %), 16.6 %^a^				*N* = 916/6465 (14.2 %), 13.6 %^a^				*N* = 2846/15,179 (18.8 %), 18.2 %^a^			
		No.^b^	(%)	%^a^	*p*	No.^b^	(%)	%^a^	*p*	No.^b^	(%)	%^a^	*p*
Demographic factors													
Age (years)													
	Young adults (19–44)	1181/7243	(16.3)	15.9	0.002	299/2190	(13.7)	13.8	0.677	882/5053	(17.5)	17.0	0.001
	Middle aged (45–64)	1500/8116	(18.5)	17.3		356/2448	(14.5)	13.5		1144/5668	(20.2)	19.3	
	Elderly (≥65)	1081/6285	(17.2)	17.1		261/1827	(14.3)	13.3		820/4458	(18.4)	18.7	
Socioeconomic factors													
Education													
	Primary or less	1290/7213	(17.9)	17.6	<.0001	181/1391	(13.0)	12.6	0.186	1109/5822	(19.1)	18.8	<.0001
	Middle school	571/2844	(20.1)	19.1		156/963	(16.2)	15.2		415/1881	(22.1)	21.0	
	High school	1066/6109	(17.5)	17.2		281/1995	(14.1)	13.9		785/4114	(19.1)	18.9	
	College or more	835/5478	(15.2)	14.8		298/2116	(14.1)	13.2		537/3362	(16.0)	15.9	
Marital status													
	Married	2384/13,602	(17.5)	16.2	0.393	569/4282	(13.3)	12.1	0.001	1815/9320	(19.5)	18.3	0.001
	Separated/divorced	897/5125	(17.5)	18.9		130/953	(13.6)	14.1		767/4172	(18.4)	20.1	
	Unmarried	481/2917	(16.5)	15.9		217/1230	(17.6)	16.6		264/1687	(15.7)	15.5	
Income													
	Quartile1	971/5376	(18.1)	19.2	0.002	253/1641	(15.4)	16.6	0.031	718/3735	(19.2)	20.5	0.024
	Quartile2	1003/5423	(18.5)	17.5		229/1571	(14.6)	13.8		774/3852	(20.1)	19.3	
	Quartile3	921/5406	(17.0)	15.9		233/1584	(14.7)	13.7		688/3822	(18.0)	17.0	
	Quartile4	867/5439	(15.9)	15.4		201/1669	(12.0)	11.9		666/3770	(17.7)	17.2	
Residence													
	Metropolitan	1177/7173	(16.4)	16.4	0.025	274/2104	(13.0)	13.1	0.150	903/5069	(17.8)	18.0	0.109
	Urban	1124/6225	(18.1)	17.3		285/1885	(15.1)	14.8		839/4340	(19.3)	18.6	
	Rural	1461/8246	(17.7)	16.1		357/2476	(14.4)	12.4		1104/5770	(19.1)	17.9	
Employment status													
	Unemployed	2250/11,138	(20.2)	20.1	<.0001	467/2492	(18.7)	19.7	<.0001	1783/8646	(20.6)	20.2	<.0001
	Employed	1512/10,506	(14.4)	13.3		449/3973	(11.3)	10.4		1063/6533	(16.3)	15.5	
Health-behaviour factors													
Smoking status													
	Current smoker	667/4313	(15.5)	15.3	<.0001	392/3146	(12.5)	12.4	0.000	275/1167	(23.6)	24.6	<.0001
	Former smoker	454/2811	(16.2)	16.6		325/2168	(15.0)	15.2		129/643	(20.1)	21.0	
	Non-smoker	2641/14,520	(18.2)	17.1		199/1151	(17.3)	14.3		2442/13,369	(18.3)	17.4	
Drinking status													
	Drinker	2732/16,737	(16.3)	15.7	<.0001	785/5874	(13.4)	12.9	<.0001	1947/10,863	(17.9)	17.3	<.0001
	Non-drinker	1030/4907	(21.0)	21.5		131/591	(22.2)	22.6		899/4316	(20.8)	21.3	
Physical activity													
	Yes	2,788/16,174	(17.2)	16.6	0.348	664/4732	(14.0)	13.5	0.631	2124/11,442	(18.6)	18.1	0.315
	No	974/5470	(17.8)	16.9		252/1733	(14.5)	13.9		722/3737	(19.3)	18.6	
Sleep hours per day													
	<6	1246/6071	(20.5)	19.6	<.0001	268/1637	(16.4)	16.0	<.0001	978/4434	(22.1)	21.2	<.0001
	≤6–9	2185/14,180	(15.4)	14.9		541/4343	(12.5)	12.0		1644/9837	(16.7)	16.4	
	≥9	331/1393	(23.8)	24.2		107/485	(22.1)	22.1		224/908	(24.7)	25.4	
Health-condition factors													
Subjective health status													
	Good	444/4163	(10.7)	10.5	<.0001	141/1500	(9.4)	9.7	<.0001	303/2663	(11.4)	11.1	<.0001
	Moderate	1119/7864	(14.2)	13.8		236/2168	(10.9)	10.6		883/5696	(15.5)	15.2	
	Poor	2199/9617	(22.9)	23.8		539/2797	(19.3)	20.0		1660/6820	(24.3)	25.5	
Perceived stress susceptibility													
	Very high	979/4168	(23.5)	23.4	<.0001	275/1373	(20.0)	20.9	<.0001	704/2795	(25.2)	24.9	<.0001
	High	1918/10,176	(18.9)	17.7		437/2871	(15.2)	14.2		1481/7305	(20.3)	19.4	
	Low	741/6370	(11.6)	11.0		175/1919	(9.1)	7.9		566/4451	(12.7)	12.4	
	Very low	124/930	(13.3)	14.1		29/302	(9.6)	8.0		95/628	(15.1)	17.2	
Unmet healthcare needs													
	Yes	1116/6865	(16.3)	16.2	0.003	251/1874	(13.4)	13.3	0.270	865/4991	(17.3)	17.6	0.002
	No	2646/14,779	(17.9)	16.8		665/4591	(14.5)	13.7		1981/10188	(19.4)	18.4	
Hypertension													
	Yes	1164/6064	(19.2)	18.9	<.0001	294/1818	(16.2)	16.0	0.004	870/4246	(20.5)	20.5	0.001
	No	2598/15,580	(16.7)	16.0		622/4647	(13.4)	12.8		1976/10,933	(18.1)	17.6	
Diabetes mellitus													
	Yes	497/2618	(19.0)	18.6	0.023	123/881	(14.0)	13.8	0.890	374/1737	(21.5)	21.4	0.002
	No	3265/19,026	(17.2)	16.4		793/5584	(14.2)	13.6		2472/13,442	(18.4)	17.8	
Hyperlipidaemia													
	Yes	845/3479	(24.3)	23.0	<.0001	190/965	(19.7)	19.5	<.0001	655/2514	(26.1)	24.7	<.0001
	No	2917/18,165	(16.1)	15.5		726/5500	(13.2)	12.5		2191/12,665	(17.3)	17.0	
CVD^c^													
	Yes	419/1933	(21.7)	21.8	<.0001	139/757	(18.4)	18.9	0.001	280/1176	(23.8)	24.1	<.0001
	No	3343/19,711	(17.0)	16.3		777/5708	(13.6)	13.1		2566/14,003	(18.3)	17.8	
Arthritis													
	Yes	1125/5119	(22.0)	22.8	<.0001	142/747	(19.0)	18.5	<.0001	983/4372	(22.5)	23.7	<.0001
	No	2637/16,525	(16.0)	15.4		774/5718	(13.5)	13.1		1863/10,807	(17.2)	16.7	
Asthma													
	Yes	282/1164	(24.2)	24.9	<.0001	70/325	(21.5)	21.4	0.000	212/839	(25.3)	26.3	<.0001
	No	3480/20,480	(17.0)	16.2		846/6140	(13.8)	13.2		2634/14,340	(18.4)	17.7	

^a^Weighted %

^b^The values were expressed as the number of participants who consulted/participants of each category

^c^CVD refers to cardiovascular disease, including angina, myocardial infarction, and stroke

Factors associated with utilization of professional mental health services for potential depression symptoms by gender are shown in Table 2. The total ORs by gender indicate that men (OR = 0.73; 95 % CI: 0.63 − 0.85) used mental health services for potential depression symptoms less frequently than did women. Both elderly men (OR = 0.67; 95 % CI: 0.54 − 0.83) and women (OR = 0.69; 95 % CI: 0.58 − 0.83) showed significantly lower rates of professional mental health service use. Regarding socioeconomic factors, women with primary education or lower (OR = 0.80; 95 % CI: 0.64 − 0.99) and both, employed men (OR = 0.55; 95 % CI: 0.45 − 0.67) and women (OR = 0.81; 95 % CI: 0.72 − 0.91) were significantly less likely to seek consultation. In terms of health behaviours, drinkers were less likely to seek consultation. Unmet healthcare needs in both genders and a history of diabetes mellitus in men were negatively associated with professional mental health service use for potential depression symptoms.

**Table 2:** Factors associated with professional mental health consultation for potential depression symptoms by gender

Characteristics		Total (*N* = 21,644)			Men (*N* = 6,465)			Women (*N* = 15,179)		
		OR	95 % CI		OR	95 % CI		OR	95 % CI	
Demographic factors										
Age (years)										
	Young adults (19–44)	1.20	1.05	1.37	1.13	0.87	1.46	1.22	1.04	1.42
	Middle aged (45–64)	1.00			1.00			1.00		
	Elderly (≥65)	0.67	0.58	0.78	0.67	0.54	0.83	0.69	0.58	0.83
Socioeconomic factors										
Education										
	Primary or less	0.81	0.67	0.97	0.77	0.58	1.02	0.80	0.64	0.99
	Middle school	1.07	0.90	1.29	1.09	0.81	1.46	1.04	0.84	1.29
	High school	1.09	0.96	1.24	1.07	0.87	1.32	1.06	0.91	1.24
	College or more	1.00			1.00			1.00		
Marital status										
	Married	0.95	0.82	1.10	0.72	0.56	0.93	1.20	1.00	1.45
	Separated/divorced	1.05	0.87	1.25	0.80	0.58	1.11	1.29	1.04	1.59
	Unmarried	1.00			1.00			1.00		
Income										
	Quartile1	0.93	0.79	1.09	0.83	0.64	1.08	0.94	0.78	1.12
	Quartile2	0.94	0.82	1.08	0.86	0.66	1.11	0.96	0.82	1.12
	Quartile3	0.97	0.86	1.11	1.10	0.88	1.36	0.92	0.80	1.07
	Quartile4	1.00			1.00			1.00		
Residence										
	Metropolitan	1.00			1.00			1.00		
	Urban	1.09	0.99	1.21	1.23	1.03	1.47	1.04	0.93	1.17
	Rural	1.01	0.90	1.13	1.02	0.85	1.22	1.01	0.89	1.14
Employment status										
	Unemployed	1.00			1.00			1.00		
	Employed	0.71	0.64	0.79	0.55	0.45	0.67	0.81	0.72	0.91
Health-behaviour factors										
Smoking status										
	Current smoker	1.09	0.94	1.27	0.86	0.70	1.06	1.41	1.18	1.68
	Former smoker	1.26	1.06	1.49	1.18	0.94	1.48	1.16	0.89	1.49
	Non-smoker	1.00			1.00			1.00		
Drinking status										
	Drinker	0.75	0.66	0.84	0.57	0.45	0.74	0.80	0.70	0.91
	Non-drinker	1.00			1.00			1.00		
Physical activity										
	Yes	1.00			1.00			1.00		
	No	0.88	0.79	0.99	0.86	0.70	1.04	0.90	0.79	1.02
Sleep hours per day										
	<6	1.17	1.06	1.30	1.28	1.07	1.53	1.14	1.02	1.28
	≤6–9	1.00			1.00			1.00		
	≥9	1.53	1.29	1.81	1.53	1.19	1.98	1.53	1.26	1.87
Health-condition factors										
Subjective health status										
	Good	1.00			1.00			1.00		
	Moderate	1.28	1.11	1.48	1.08	0.86	1.38	1.38	1.17	1.64
	Poor	2.31	1.97	2.70	1.99	1.54	2.56	2.48	2.06	2.98
Perceived stress susceptibility										
	Very high	1.69	1.26	2.27	3.08	1.85	5.14	1.36	0.98	1.89
	High	1.27	0.95	1.69	2.05	1.23	3.41	1.09	0.80	1.49
	Low	0.77	0.57	1.04	1.09	0.65	1.85	0.69	0.50	0.96
	Very low	1.00			1.00			1.00		
Unmet healthcare needs										
	Yes	0.78	0.71	0.87	0.77	0.63	0.93	0.79	0.70	0.88
	No	1.00			1.00			1.00		
Medical history										
	Hypertension	1.00	0.88	1.13	1.12	0.92	1.37	0.95	0.83	1.10
	Diabetes mellitus	0.81	0.70	0.95	0.67	0.53	0.86	0.89	0.75	1.07
	Hyperlipidaemia	1.47	1.30	1.66	1.65	1.34	2.04	1.42	1.24	1.63
	CVD^a^	1.06	0.90	1.24	1.02	0.81	1.29	1.06	0.87	1.29
	Arthritis	1.35	1.19	1.53	1.24	0.94	1.62	1.36	1.19	1.56
	Asthma	1.32	1.10	1.58	1.36	0.99	1.88	1.30	1.07	1.59

OR refers to odds ratio, and 95 % CI refers to 95 % confidence interval

^a^CVD refers to cardiovascular disease, including angina, myocardial infarction, and stroke

Table 3 shows professional mental health consultation rates in men by age. Young adult (OR = 0.39; 95 % CI: 0.18 − 0.84) and elderly men (OR = 0.70; 95 % CI: 0.54 − 0.90) with the lowest education level had significantly lower rates of professional mental health service use than did those with the highest education level. Among elderly men, rates of utilization for those in the lowest (OR = 0.36; 95 % CI: 0.29 − 0.45) and second lowest (OR = 0.43; 95 % CI: 0.34 − 0.54) income quartiles differed significantly from those in the highest quartile. With respect to health-related factors, elderly current smokers vs. non-smokers (OR = 0.54; 95 % CI: 0.41 − 0.70) and drinkers vs. non-drinkers (OR = 0.65; 95 % CI: 0.52 − 0.80) were significantly less likely to seek consultation. Middle-aged individuals with unmet healthcare needs had a significantly lower consultation rate than did those who did not have unmet needs (OR = 0.73; 95 % CI: 0.58 − 0.92). Low perceived stress susceptibility among middle-aged individuals was associated with a lower consultation rate (OR = 0.64; 95 % CI: 0.43 − 0.96), whereas young adults with very high perceived stress susceptibility had higher consultation rates (OR = 5.05; 95 % CI: 3.48 − 7.32). Middle-aged individuals with a history of diabetes mellitus (OR = 0.62; 95 % CI: 0.47 − 0.82) and asthma (OR = 0.65; 95 % CI: 0.47 − 0.91) had lower consultation rates, whereas among elderly individuals, those with asthma had higher consultation rates (OR = 2.94; 95 % CI: 2.46 − 3.53).

**Table 3:** Factors associated with consultation rates for potential depression symptoms in men after stratification by age

Characteristics		Young adults			Middle aged			Elderly		
		(19–44 years)			(45–64 years)			(≥65 years)		
		OR	95 % CI		OR	95 % CI		OR	95 % CI	
Socioeconomic factors										
Education										
	Primary or less	0.39	0.18	0.84	0.73	0.51	1.06	0.70	0.54	0.90
	Middle school	1.10	0.50	2.41	1.14	0.85	1.51	0.86	0.62	1.18
	High school	1.09	0.86	1.37	0.87	0.66	1.15	1.22	0.96	1.54
	College or more	1.00			1.00			1.00		
Marital status										
	Married	0.78	0.63	0.96	0.48	0.30	0.75	0.62	0.41	0.93
	Separated/divorced	0.87	0.50	1.53	0.56	0.35	0.89	0.57	0.37	0.89
	Unmarried	1.00			1.00			1.00		
Income										
	Quartile1	1.14	0.80	1.63	0.69	0.50	0.96	0.36	0.29	0.45
	Quartile2	0.95	0.67	1.33	0.84	0.62	1.15	0.43	0.34	0.54
	Quartile3	1.23	0.99	1.52	0.95	0.72	1.24	0.80	0.62	1.03
	Quartile4	1.00			1.00			1.00		
Residence										
	Metropolitan	1.00			1.00			1.00		
	Urban	0.99	0.80	1.22	1.40	1.14	1.72	1.56	1.29	1.89
	Rural	0.79	0.61	1.02	0.97	0.77	1.22	1.75	1.44	2.11
Employment status										
	Unemployed	1.00			1.00			1.00		
	Employed	0.62	0.48	0.79	0.38	0.30	0.49	0.94	0.75	1.18
Health-behaviour factors										
Smoking status										
	Current smoker	0.80	0.63	1.01	1.05	0.80	1.38	0.54	0.41	0.70
	Former smoker	1.32	0.96	1.81	1.08	0.82	1.42	1.09	0.86	1.38
	Non-smoker	1.00			1.00			1.00		
Drinking status										
	Drinker	0.39	0.28	0.56	0.83	0.60	1.13	0.65	0.52	0.80
	Non-drinker	1.00			1.00			1.00		
Physical activity										
	Yes	1.00			1.00			1.00		
	No	1.02	0.78	1.34	0.82	0.63	1.06	0.65	0.52	0.77
Sleep hours per day										
	<6	1.27	1.02	1.58	1.31	1.07	1.60	1.43	1.18	1.72
	≤6–9	1.00			1.00			1.00		
	≥9	1.51	1.05	2.17	1.37	1.01	1.86	1.83	1.42	2.35
Health-condition factors										
Subjective health status										
	Good	1.00			1.00			1.00		
	Moderate	0.96	0.75	1.23	1.26	0.94	1.68	1.58	0.85	2.96
	Poor	1.60	1.18	2.16	2.04	1.49	2.78	3.41	1.79	6.49
Perceived stress susceptibility										
	Very high	5.05	3.48	7.32	1.87	1.30	2.67	3.05	2.00	4.64
	High	3.59	2.54	5.09	1.07	0.75	1.52	2.27	1.50	3.45
	Low	1.65	1.14	2.38	0.64	0.43	0.96	1.17	0.74	1.85
	Very low	1.00			1.00			1.00		
Unmet healthcare needs										
	Yes	0.78	0.61	1.00	0.73	0.58	0.92	0.84	0.68	1.04
	No	1.00			1.00			1.00		
Medical history										
	Hypertension	1.46	1.05	2.04	1.03	0.84	1.27	1.06	0.89	1.25
	Diabetes mellitus	0.58	0.32	1.05	0.62	0.47	0.82	0.88	0.74	1.06
	Hyperlipidaemia	1.57	1.16	2.13	1.94	1.55	2.42	1.21	0.99	1.48
	CVD^a^	3.19	1.91	5.33	0.91	0.71	1.17	0.84	0.69	1.03
	Arthritis	1.38	0.90	2.11	0.93	0.64	1.36	1.46	1.23	1.72
	Asthma	1.09	0.79	1.51	0.65	0.47	0.91	2.94	2.46	3.53

OR refers to odds ratio and 95 % CI refers to 95 % confidence interval

^a^CVD refers to cardiovascular disease, including angina, myocardial infarction, and stroke

Table 4 presents rates of professional mental health consultation for potential depression symptoms in women by age. Only among elderly women were professional mental health consultation rates were significantly lower among those with the lowest education level, compared to those with the highest education level (OR = 0.53; 95 % CI: 0.36 − 0.77). Young adults in the lowest income quartile were significantly more likely to seek consultation (OR = 1.61; 95 % CI: 1.19 − 2.18), whereas elderly individuals in the lowest quartile were significantly less likely to seek consultation (OR = 0.62; 95 % CI: 0.49 − 0.79). With respect to health-related factors, elderly individuals who were former smokers had lower consultation rates (OR = 0.72; 95 % CI: 0.52 − 0.99). Drinkers had significantly lower consultation rates than did non-drinkers among both young adults (OR = 0.57; 95 % CI: 0.45 − 0.72) and middle-aged individuals (OR = 0.79; 95 % CI: 0.67 − 0.92). Low perceived stress susceptibility was associated with lower consultation rates only among elderly individuals (OR = 0.59; 95 % CI: 0.38 − 0.94). Middle-aged (OR = 0.78; 95 % CI: 0.67 − 0.91) and elderly individuals (OR = 0.66; 95 % CI: 0.55 − 0.78) with unmet healthcare needs had significantly lower consultation rates than did those without unmet needs. A history of hypertension (OR = 0.75; 95 % CI: 0.65 − 0.86) and diabetes mellitus (OR = 0.82; 95 % CI: 0.67 − 0.99) were associated with lower rates of consultation, but only among elderly individuals.

**Table 4:** Factors associated with consultation rates for potential depression symptoms in women after stratification by age

Characteristics		Young adults			Middle aged			Elderly		
		(19–44 years)			(45–64 years)			(≥65 years)		
		OR	95 % CI		OR	95 % CI		OR	95 % CI	
Socioeconomic factors										
Education										
	Primary or less	0.95	0.52	1.75	0.81	0.63	1.05	0.53	0.36	0.77
	Middle school	0.90	0.64	1.27	0.94	0.73	1.20	0.74	0.50	1.10
	High school	1.08	0.92	1.28	0.86	0.68	1.09	1.00	0.64	1.56
	College or more	1.00			1.00			1.00		
Marital status										
	Married	1.15	0.96	1.38	2.24	1.06	4.73	1.13	0.65	1.98
	Separated/divorced	1.24	0.90	1.70	2.29	1.09	4.81	1.26	0.72	2.20
	Unmarried	1.00			1.00			1.00		
Income										
	Quartile1	1.61	1.19	2.18	0.97	0.77	1.22	0.62	0.49	0.79
	Quartile2	1.04	0.83	1.31	0.82	0.68	0.99	0.81	0.63	1.05
	Quartile3	0.98	0.82	1.18	0.84	0.69	1.03	0.80	0.61	1.05
	Quartile4	1.00			1.00			1.00		
Residence										
	Metropolitan	1.00			1.00			1.00		
	Urban	0.98	0.83	1.15	1.10	0.95	1.28	1.09	0.91	1.30
	Rural	0.89	0.72	1.10	1.05	0.88	1.25	1.17	0.98	1.40
Employment status										
	Unemployed	1.00			1.00			1.00		
	Employed	0.81	0.69	0.94	0.81	0.70	0.94	0.85	0.66	1.09
Health-behaviour factors										
Smoking status										
	Current smoker	1.68	1.34	2.12	1.40	1.10	1.78	0.76	0.52	1.10
	Former smoker	1.52	1.10	2.11	0.98	0.72	1.34	0.72	0.52	0.99
	Non-smoker	1.00			1.00			1.00		
Drinking status										
	Drinker	0.57	0.45	0.72	0.79	0.67	0.92	1.01	0.86	1.19
	Non-drinker	1.00			1.00			1.00		
Physical activity										
	Yes	1.00			1.00			1.00		
	No	0.98	0.80	1.18	0.92	0.78	1.07	0.82	0.69	0.97
Sleep hours per day										
	<6	1.07	0.80	1.29	1.15	0.99	1.33	1.18	1.01	1.38
	≤6–9	1.00			1.00			1.00		
	≥9	1.49	1.13	1.95	2.01	1.46	2.77	1.10	0.84	1.44
Health-condition factors										
Subjective health status										
	Good	1.00			1.00			1.00		
	Moderate	1.33	1.10	1.61	1.39	1.10	1.76	1.45	0.94	2.25
	Poor	2.37	1.89	2.97	2.44	1.88	3.16	2.72	1.76	4.19
Perceived stress susceptibility										
	Very high	1.70	0.92	3.16	1.15	0.75	1.75	1.55	0.93	2.58
	High	1.39	0.76	2.55	1.02	0.68	1.53	1.04	0.67	1.63
	Low	0.78	0.42	1.45	0.71	0.46	1.10	0.59	0.38	0.94
	Very low	1.00			1.00			1.00		
Unmet healthcare needs										
	Yes	0.89	0.76	1.05	0.78	0.67	0.91	0.66	0.55	0.78
	No	1.00			1.00			1.00		
Medical history										
	Hypertension	1.42	0.94	2.16	1.01	0.86	1.18	0.75	0.65	0.86
	Diabetes mellitus	1.00	0.64	1.55	0.95	0.77	1.17	0.82	0.67	0.99
	Hyperlipidaemia	1.68	1.24	2.28	1.12	0.96	1.31	1.81	1.54	2.13
	CVD^a^	0.44	0.23	0.84	1.34	1.07	1.68	0.93	0.77	1.13
	Arthritis	1.56	1.15	2.12	1.40	1.21	1.62	1.26	1.08	1.46
	Asthma	1.40	1.00	1.96	1.36	1.04	1.77	1.22	0.96	1.55

OR refers to odds ratio and 95 % CI refers to 95 % confidence interval

^a^CVD refers to cardiovascular disease, including angina, myocardial infarction, and stroke

## Discussion

We investigated demographic, socioeconomic, and health-related, and health-condition factors associated with the underutilization of professional mental health services for potential depression symptoms. Among those who reported depression symptoms during the past year, we observed that 17.4 % had received professional mental health consultation for the depression symptoms. Young adults showed the lowest prevalence of professional mental health service use, but after adjustment of demographic, socioeconomic, and health-related factors, we observed the higher rate of service use, which is inconsistent with a previous study showing more negative attitudes in young and middle-aged adults [7].

A previous study found that elderly individuals with depression were less likely to contact mental health professionals than middle-aged individuals were [7], which is consistent with our findings. Although protective factors—such as high self-reliance—in older adults could be linked to underutilization of mental health services [7, 23], elderly individuals with potential depression symptoms are more likely to have co-existing chronic illnesses, such as cardiovascular disease [24, 25], or functional disability [26]. In addition, prior to committing suicide, elderly individuals tend to contact primary care providers rather than psychiatrists [27]. Collectively, these findings highlight the role of primary care providers in the management of mental health of elderly individuals [28, 29].

The present study showed that men contacted mental health professionals for potential depression symptoms less frequently than women did, which is consistent with previous findings on mental health service use [7, 30]. This may be related to gender differences in help-seeking behaviours [31]. Specifically, men may perceive greater stigma, while women may more easily acknowledge and recognize mental illnesses from nonspecific distressful emotions [32, 33]. Contrastingly, because women tend to internalise their feelings [34], they may have more severe symptoms, which is consistent with the significantly larger proportion of women with high perceived stress in our study.

Although low socioeconomic status may increase the risk for depression and suicidal ideation/behaviours [35–37], the present study showed no significant difference in the rates of seeking formal mental healthcare for potential depression symptoms across income quartiles. This may be attributable to the National Health Insurance system adopted in the Republic of Korea, which may have helped reduce the socioeconomic gap in medical care utilization [38]. However, after stratification by age, young adults with the lowest income level showed higher rates for mental health service use compared to all other income groups, while elderly individuals with the lowest income level had lower consultation rates compared to the highest income level. The effect modification of age may indicate that elderly individuals’ use of mental health services depends more on income than it does for other age groups. In the Republic of Korea, elderly individuals’ suicidal behaviours are most likely motivated by financial problems [39]. Further, the income poverty rates late 2000s for elderly Koreans was 45.6 %, which is four times higher than the average among OECD countries [40]. Although elderly people with financial problems may be exposed to greater psychosocial distress, they were less likely to seek professional help for potential depression symptoms. Further, elderly individuals with a lower income may be socially isolated and may have difficulties engaging in help-seeking behaviours, even though they show positive help-seeking attitudes for mental health problems. Thus, it is imperative that target strategies for elderly individuals’ mental health are established at the community level, such as strengthening social networks.

Although unemployment is associated with suicide [41, 42], the present study suggests that employed individuals were less likely to use professional mental health services than unemployed individuals were. One explanation is the stigma surrounding depressive disorders [43]. In this regard, the Korean government plans to revise the Mental Health Act such that patients with mild symptoms—requiring only outpatient care—will be excluded from the definition of a ‘psychiatric patient’ [44]. However, this change may not address the psychosocial aspects of stigma; interventions that strengthen social supports and discourage discrimination against individuals with mental illnesses need to be enforced. Nonetheless, our findings should be interpreted with caution because we did not consider time-dependent employment status. Those who were unemployed at the time of survey completion may have been previously employed and may have lost their jobs as a result of severe depressive symptoms. In this regard, programs in the workplace for employees’ mental health can help prevent the aggravation of depression symptoms.

Patients with diabetes mellitus were significantly less likely to seek consultation for potential depression symptoms, whereas those with other chronic diseases, such as hypertension, had high consultation rates. Diabetes increases the risk for psychosocial problems, which may have an adverse effect on diabetic patients’ self-care practices [45]. A previous study reported that approximately 10 % of patients with diabetes and psychosocial problems received professional help for their psychosocial problems [45]. One explanation for the discrepancy between diabetes and other chronic diseases is that patients with diabetes and psychosocial problems are less likely to engage in effective diabetes management because of the less noticeable symptoms even with poor management (e.g. pain or dyspnea in patients with mild diabetes). However, we were unable to classify participants according to the severity of diabetes mellitus in the present study. Further investigation of the link between diabetes, psychological problems, and mental health service utilization needs to be conducted.

### Strengths and limitations

In this study, we used a large and nationally representative sample to investigate the factors associated with utilization rates of professional mental health consultation for potential depression symptoms. However, several limitations should be considered. Healthy individuals were included among those asked to complete the CHS questionnaires. Thus, the survey did not exclusively focus on those with severe mental problems or suicidal completion. However, given that the present study intended to explore factors associated with underutilization of mental health services, the exclusion of severe cases from this study might not have distorted our results. The participants were selected based on a single self-reported item on depressive symptoms, rather than assessments using psychometric instruments. However, this question is used to determine an essential criterion for major depressive disorder as per the Diagnostic and Statistical Manual of Mental Disorders, 4^th^ Edition. Thus, regardless of diagnostic criteria for specific disorders, we were able to identify individuals with depressive symptoms who deserve further evaluation by mental health professionals. Moreover, even though participants who reported having consulted a mental health professional may not have met the criteria for depressive disorders, this does not diminish the present findings since our focus was on patients who required mental health consultation rather than those who met the criteria for depressive disorders. Furthermore, we used a cross-sectional design, which precludes any inferences regarding causation (i.e. the possibility of reverse causality remains). Thus, any interpretation of time-dependent variables (i.e. marital, employment, smoking, and drinking status) should be considered with caution; future studies utilizing a prospective design are needed.

## Conclusions

In the present study, among those who reported potential depression symptoms, only 17.4 % had consulted a mental health professional for their symptoms. Elderly individuals were less likely to contact mental health professionals for potential depression symptoms compared to middle-aged individuals. In particular, elderly individuals in the lowest income group were less likely to use mental health services compared to the highest income group. To develop prevention and management strategies for elderly individuals with potential depression symptoms, it is necessary to underscore the role of primary care providers and establish target strategies at the community level, such as strengthening social networks.

### Ethical standards

The authors assert that all procedures contributing to this work comply with the ethical standards of the relevant national and institutional committees on human experimentation and with the Helsinki Declaration of 1975, as revised in 2008. However, no additional permissions were required since public access data were used.

## Additional file

Additional file 1: Table S1. Total participants in the Community Health Survey (*N* = 458,147). (DOC 83 kb)

## Abbreviations

CHS: Community Health Survey
CVD: Cardiovascular disease
OR: Odds ratio
CI: Confidence interval
OECD: Organisation for Economic Co-operation and Development
