# Supplementary material for: Depression symptom and professional mental health service use
Source: BMC Psychiatry. 2015 Oct 24;15:261. doi: 10.1186/s12888-015-0646-z (PMC4619991; doi:10.1186/s12888-015-0646-z)
Supplement: Additional file 1: Table S1. — Total participants in the Community Health Survey (N = 458,147). (DOC 83 kb) [file 12888_2015_646_MOESM1_ESM.doc]

**Additional file 1: Table S1 Total participants in the Community Health Survey (N = 458,147).**

| **Characteristics** | | **N** | **%** | **%*** |  | **Characteristics** | | **N** | **%** | **%*** |
| --- | --- | --- | --- | --- | --- | --- | --- | --- | --- | --- |
| Age, mean (SD) | | 51.22 (16.89) | | |  | Stress | |  |  |  |
| Gender | |  |  |  |  |  | Very high | 15,241 | 3.3 | 3.7 |
|  | Men | 205,915 | 45.0 | 49.5 |  |  | High | 105,941 | 23.1 | 24.7 |
|  | Women | 252,232 | 55.1 | 50.5 |  |  | Low | 243,835 | 53.2 | 54.9 |
| Education | |  |  |  |  |  | Very low | 92,529 | 20.2 | 16.5 |
|  | Primary or less | 122,041 | 26.6 | 14.2 |  |  | Missing | 601 | 0.1 | 0.1 |
|  | Middle school | 53,328 | 11.6 | 9.2 |  | Unmet healthcare needs | | |  |  |
|  | High school | 133,489 | 29.1 | 31.0 |  |  | Yes | 60,003 | 13.1 | 12.9 |
|  | College or more | 148,386 | 32.4 | 45.3 |  |  | No | 398,024 | 86.9 | 87.1 |
|  | Missing | 903 | 0.2 | 0.2 |  |  | Missing | 120 | 0.0 | 0.0 |
| Marital status | |  |  |  |  | Hypertension | |  |  |  |
|  | Married | 326,905 | 71.4 | 68.2 |  |  | Yes | 104,996 | 22.9 | 17.3 |
|  | Separated/divorced | 64,658 | 14.1 | 9.7 |  |  | No | 352,986 | 77.1 | 82.7 |
|  | Unmarried | 66,235 | 14.5 | 22.0 |  |  | Missing | 165 | 0.0 | 0.0 |
|  | Missing | 349 | 0.1 | 0.1 |  | Diabetes mellitus | |  |  |  |
| Income, mean (SD) | | 7041.25 (22126) | | |  |  | Yes | 39,314 | 8.6 | 6.5 |
| Residence | |  |  |  |  |  | No | 418,590 | 91.4 | 93.4 |
|  | Metropolitan | 135,773 | 29.6 | 46.3 |  |  | Missing | 243 | 0.1 | 0.1 |
|  | Urban | 129,223 | 28.2 | 35.7 |  | Hyperlipidaemia | |  |  |  |
|  | Rural | 193,151 | 42.2 | 17.9 |  |  | Yes | 46,777 | 10.2 | 9.8 |
| Employment status | |  |  |  |  |  | No | 410,391 | 89.6 | 90.0 |
|  | Unemployed | 169,345 | 37.0 | 36.4 |  |  | Missing | 979 | 0.2 | 0.2 |
|  | Employed | 288,251 | 62.9 | 63.4 |  | CVD‡ | |  |  |  |
|  | Missing | 551 | 0.1 | 0.2 |  |  | Yes | 20,780 | 4.5 | 3.2 |
| Smoking status | |  |  |  |  |  | No | 437,265 | 95.4 | 96.8 |
|  | Current smoker | 96,491 | 21.1 | 23.8 |  |  | Missing | 102 | 0.0 | 0.0 |
|  | Ex-smoker | 73,443 | 16.0 | 15.6 |  | Arthritis | |  |  |  |
|  | Non-smoker | 288,135 | 62.9 | 60.5 |  |  | Yes | 61,591 | 13.4 | 8.5 |
|  | Missing | 78 | 0.0 | 0.0 |  |  | No | 396,278 | 86.5 | 91.4 |
| Drinking status | |  |  |  |  |  | Missing | 278 | 0.1 | 0.0 |
|  | Drinker | 358,076 | 78.2 | 15.3 |  | Asthma | |  |  |  |
|  | Non-drinker | 100,031 | 21.8 | 84.7 |  |  | Yes | 12,497 | 2.7 | 2.4 |
|  | Missing | 40 | 0.0 | 0.0 |  |  | No | 445,497 | 97.2 | 97.6 |
| Physical activity | |  |  |  |  |  | Missing | 153 | 0.0 | 0.0 |
|  | Yes | 348,749 | 76.1 | 18.0 |  | Depression symptoms | |  |  |  |
|  | No | 108,709 | 23.7 | 81.9 |  |  | Yes | 23,625 | 5.2 | 5.4 |
|  | Missing | 689 | 0.2 | 0.1 |  |  | No | 434,209 | 94.8 | 94.5 |
| Sleep hours per day, mean (SD) | | 6.76 (3.17) | | |  |  | Missing | 313 | 0.1 | 0.1 |
| Subjective health status | |  |  |  |  | Mental health consultation for depression symptoms | | | | |
|  | Good | 180,191 | 39.3 | 44.0 |  |  | Yes | 4,083 | 0.9 | 4.5 |
|  | Moderate | 180,054 | 39.3 | 41.0 |  |  | No | 19,541 | 4.3 | 0.9 |
|  | Poor | 97,840 | 21.4 | 15.0 |  |  | Missing^§^ | 434,523 | 94.8 | 94.6 |
|  | Missing | 62 | 0.0 | 0.0 |  |  |  |  |  |  |
| SD refers to standard deviation. | | | | | | | | | | |
| ‡ CVD refers to cardiovascular disease, including angina, myocardial infarction, and stroke. | | | | | | | | | | |
| § Including the participants without depression symptoms. | | | | | | | | | | |
